# Marker assisted pyramiding of *Bph6* and *Bph9* into elite restorer line 93–11 and development of functional marker for *Bph9*

**DOI:** 10.1186/s12284-017-0194-x

**Published:** 2017-12-28

**Authors:** Yang Wang, Weihua Jiang, Hongmei Liu, Ya Zeng, Bo Du, Lili Zhu, Guangcun He, Rongzhi Chen

**Affiliations:** State Key Laboratory of Hybrid Rice, College of Life Sciences, Wuhan University, Wuhan, 430072 China

**Keywords:** Rice, Brown planthopper (BPH), Marker-assisted selection (MAS), Pyramiding breeding, Functional marker (FM)

## Abstract

**Background:**

The brown planthopper (BPH) has become the most destructive and a serious threat to the rice production in Asia. Breeding the resistant varieties with improved host resistance is the most effective and ecosystem-friendly strategy of BPH biological management. As host resistance was always broken down by the presence of the upgrading BPH biotype, the more resistant varieties with novel resistance genes or pyramiding known identified BPH resistance genes would be needed urgently for higher resistant level and more durability of resistance.

**Results:**

Here, we developed near isogenic lines of *Bph9* (NIL-*Bph9*) by backcrossing elite cultivar 93–11 with Pokkali (harboring *Bph9*) using marker-assisted selection (MAS). Subsequently, we pyramided *Bph6* and *Bph9* in 93–11 genetic background through MAS. The resulting *Bph6* and *Bph9* pyramided line LuoYang69 had stronger antixenotic and antibiosis effects on BPH and exhibited significantly enhanced resistance to BPH than near isogenic lines NIL-*Bph6* and NIL-*Bph9*. LuoYang69 derived hybrids, harboring heterozygous *Bph6* and *Bph9* genes, also conferred high level of resistance to BPH. Furthermore, LuoYang69 did not affect the elite agronomic traits and rice grain quality of 93–11. The current study also developed functional markers for *Bph9.* Using functional dominant marker, we screened and evaluated worldwide accessions of rice germplasm. Of the 673 varieties tested, 8 cultivars were identified to harbor functional *Bph9* gene.

**Conclusion:**

The development of *Bph6* and *Bph9* pyramided line LuoYang69 provides valuable resource to develop hybrid rice with highly and durable BPH resistance. The development of functional markers will promote MAS of *Bph9.* The identified *Bph9* containing cultivars can be used as new sources for BPH resistance breeding programs.

**Electronic supplementary material:**

The online version of this article (10.1186/s12284-017-0194-x) contains supplementary material, which is available to authorized users.

## Background

Rice (*Oryza sativa* L.), an important cereal crop in Asian-Pacific region, is a stable food resource for more than half the population of the world (Du et al., 2009; Hu et al., 2016). Increasing yield has become the most core goal of rice production. Nevertheless, like the other plants, the growth of rice was disrupted by insect pests attacking, which will lead to significantly yield loss as the major biotic constraint (Myint et al., 2012). Of more than 20 kinds of serious paddy pests known at present, brown planthopper (BPH, *Nilaparvata lugens* Stål), a migratory and monophagous rice insect, has proved to be the most destructive, especially in the Asia (Sogawa et al., 2003; Brar et al., 2009). BPH not only sucks the phloem sap of rice leaf sheath, but also transmits the viral disease such as rice grassy stunt virus (RGSV), rice ragged stunt virus (RRSV) and rice wilted stunt virus (RWSV) (Heinrichs, 1979; Alam and Cohen, 1998b). Heavy infestation of BPH will result in complete dying of rice, referring the so called “hopperburn” (Watanabe and Kitagawa, 2000).

For preventing the damage caused by BPH, several approaches have been implemented including chemical and biological controlling (Normile, 2008). Recent reports showed the abuse of pesticide or insectcide wrecked the balance of the natural ecosystem by the heavily pollution and would bring about plasticity of BPH to the insecticide (Matteson, 2000; Tanaka et al., 2000; Park et al., 2007; Lakshmi et al., 2010). Comparing with the conventional chemical controlling, developing the host resistance by BPH resistance genes has been regarded as the most economic effective and environmental-friendly solution for controlling it (Matsumura et al., 2009). Seeking BPH-resistant germplasm resources from various varieties and utilizing such resistance genes has turned into the most important component of breeding programs (Pathak et al., 1969; Alam and Cohen, 1998a). To date, 31 BPH-resistant genes have been identified in the cultivated rice and wild *Oryza* species, thirteen of these resistance genes have been cloned by map-based cloning (Ji et al., 2016; Ren et al., 2016; Du et al., 2009; Tamura et al., 2014; Wang et al., 2015; Zhao et al., 2016; Liu et al. 2015; Jing et al., 2017), which provide resistance genes for marker assisted selection (MAS) breeding. However, the varieties bearing single BPH resistance gene were quickly broken down within a few years due to rapid adaptation of BPH or evolution of new biotypes (Jena and Kim, 2010). For instance, the variety of IR26 developed by IRRI with single resistance gene *Bph1* showed a moderate resistance to BPH biotype 1 in the early 1970s. However, the resistance was rapidly broken down by BPH biotype 2 after 2–3 years (Khush, 1977). It has been proposed that pyramiding multiple BPH resistance genes is an efficient strategy to develop more durable resistant varieties against BPH. Myint et al., 2012) found that the BPH resistance level of (*Bph25* + *Bph26*)-NILs was significantly higher than either *Bph25*-NILs or *Bph26*-NILs. An additive effect occurred after pyramiding three dominant BPH- resistance genes (*Bph14*, *Bph15* and *Bph18*) into the elite indica rice variety 93–11 than the double gene lines and monogenic lines (Hu et al., 2013). *Bph14*, *Bph15*, *Cry1C*, and *bar* were pyramided for an elite restorer lines with processing resistance durability to BPH (Wan et al., 2014). The *Bph3* and *Bph27(t)* single gene introgression *japonica* lines were inter-crossed following the methods of MAS, and the more durable pyramiding variety with *japonica* context was developed for crop breeding (Liu et al., 2016). Wang et al., 2016) further improved the BPH-resistance of Huahui938 and its derived hybrids by pyramiding *Bph14* and *Bph15* using molecular marker-assisted backcrossing (MAB).

The rice variety Swarnalata, carrying BPH resistance gene *Bph6* and resisting BPH biotype 4 (Bangladesh BPH population), also exhibited high level of resistance to BPH population in China (biotype 2 being the dominant one). *Bph6* in Swarnalata was fine-mapped between the STS markers Y9 and Y19 on the long arm of chromosome 4 (Qiu et al., 2010). Three Sri Lanka varieties, Kaharamana, Balamawee and Pokkali, harboring a dominant BPH resistance gene *Bph9*, were shown to be resistant to BPH biotypes 1, 2 and 3. Nemoto et al., 1989) firstly mapped *Bph9* in Pokkali between RFLP marker OPRO4 and RAPD marker S2545 on chromosome 12. Su et al., 2006) located *Bph9* in Kaharamana between SSR markers RM463 and RM5341 on chromosome 12. Recently, *Bph9* from Pokkali has been cloned, encoding a rare type of nucleotide-binding and leucine-rich repeat (NLR) gene (Zhao et al., 2016). The cloning of *Bph9* revealed that the eight BPH resistance genes clustered on the long arm of chromosome 12 are actually allelic with each other and can be classified into four allelotypes (Zhao et al., 2016).

93–11 is an elite restorer line for two-line and CMS three-line hybrid rice. In present study, we introgressed *Bph9* into 93–11 using marker-assisted selection (MAS). We further pyramided *Bph6* and *Bph9* into 93–11 through MAS. The resulting *Bph6* and *Bph9* pyramided line LuoYang69 had stronger antixenotic and antibiosis effects on BPH and exhibited significantly enhanced resistance than NIL-*Bph6* and NIL-*Bph9*. Its derived hybrids, harboring heterozygous *Bph6* and *Bph9* genes, also conferred high level of resistance to BPH. The development of LuoYang69 with *Bph6* and *Bph9* pyramided provides valuable resource to develop hybrid rice with high BPH resistance and excellent agronomic performance. The current work also developed functional markers for MAS of *Bph9* in rice breeding programs. We screened and evaluated worldwide accessions of rice germplasm with functional dominant marker. 8 cultivars of the 673 varieties tested were identified to harbor functional *Bph9* gene, providing new sources for BPH resistance breeding programs.

## Methods

### Plant materials, molecular markers and breeding strategies for NIL-*Bph9* and LuoYang69 development

93–11, an elite indica rice cultivar with good quality, high yield, while highly susceptible to BPH, was used as the recurrent paternal plant in backcrossing. The Sri Lanka rice indica cultivar, Pokkali, harboring BPH resistance gene *Bph9*, was used as the donor maternal for cross. As shown in Additional file 1: Figure S1, near isogenic lines of *Bph9* in 93–11 background was developed by successive backcrossing of the 93–11/Pokkali F_1_ with 93–11. During this process, the gene-linked markers InD2 and RM28466 flanking the *Bph9* locus were used to select plants with heterozygous *Bph9* from each backcrossed populations for the next step of backcrossing. The sequences of InD2 and RM28466 were shown as below: InD2 (F: 5’-AACAGACACGTTGCGTCTTG-3′, R: 5’-CTTGCCGCTTAGAGGAGATG-3′) RM28466 (F: 5’-CCGACGAAGAAGACG.

AGGAGTAGCC-3′, R: 5’-AGGCCGGAGAGCAATCATGTCG-3′). NIL-*Bph6* was developed by an extra generation of backcrossing BC_4_F_1_ plant 4Q1100–5-9 (Qiu et al., 2010) with 93–11. Indel marker H tightly linked with *Bph6* locus was used to select plants with heterozygous genotype of *Bph6* from the final cycle of backcrossing. The sequence of H marker was shown below: H (F: 5’-AGAATTGCTGCATGCTGTTG-3′, R: 5’-ATTCCAGCATCGATTGCTTC-3′). The *Bph6* and *Bph9* pyramided line was developed by crossing NIL-*Bph6* and NIL-*Bph9*. The F_3_ plants with homozygous *Bph9* and *Bph6* were selected, and renamed as LuoYang69. For identifying *Bph9* in core collection of germplasm and other cultivars, the functional marker B9D was designed within a 1.2 kb specific fragment in intron 1 of *Bph9* allele in Pokkali. The sequence of B9D was shown below: (F: 5’-ACGGCACGTAGACCAAAAAC-3′, R: 5’-TCGGTTGTCGAACTCTTGTC-3′). An Indel co-dominant marker IR2 was designed in intron 2 of *Bph9* allele in Pokkali for differentiate the homozygous heterozygous status of *Bph9*. The sequence of IR2 was shown as below: IR2 (F: 5’-AGGATGGGGAGAAGAAGACG-3′, R: 5’-TACACCCGACAAGGAACAC-3′). Genomic sequence and SSR markers used in RRGB test were obtained from the GRAMENE (http: //www. gramene.org/markers/index.html).

### DNA extraction and genotyping

The total genomic DNA was extracted from fresh rice leaves at three-leaf stage using modified CTAB protocol for avoiding the pollution of polysaccharide and polyphenol components (Porebski et al., 1997). The extracted DNA was dissolved in 1 × TE buffer. Targeting sequences were amplified using PCR protocols described by Yang et al., 2002), with minor modifications for different primers. The PCR products of RRGB test were analyzed by 6% denaturing polyacrylamide gel electrophoresis and visualized by silver staining modified by Qiu et al., 2010). The PCR products of MAS were checked by 1.5% agarose gel electrophoresis stained with 2 μL EB, respectively. The whole-genome single nucleotide polymorphsim (SNP) array RICE6K was used to analyze the RRGB of the pyramided line. This SNP array containing 5102 SNP and InDel markers evenly distributed on the 12 chromosomes of rice was developed based on Infinium technology (Yu et al., 2014). The RRGB analysis by RICE6K array was performed at the Life Science and Technology Center, China National Seed Group Co., LTD (Wuhan, China), according to Infinium HD Assay Ultra Protocol (http:// www.illumina.com/).

### BPH population and bioassay for evaluation of BPH resistance

The BPH populations used for resistance evaluation were collected from the field in summer at Wuhan University and were fed continuously on Tai-chung Native 1 (TN1, a susceptible indica variety) under greenhouse condition. The Bioassay for BPH resistance evaluation was performed by the a little-modified standard seedling bulk test according to Huang (Huang et al., 2001). The seeds of each rice resistance-improved varieties and susceptible varieties were pre-germinated for the identical developmental stage. Thirty seeds from individual resistance-improved plant were sown in the plastic box (30 length × 18 width × 12 cm height) in rows which were flanked by rows of seeds of the susceptible lines (TN1 or 93–11). The seedlings were thinned to 20 plants evenly distributed per row about 7 days after sowing. At third-leaf stage, the seedlings were infested with 2nd to 3rd instar BPH nymphs at a rate of 20 insects per seedling. After infestation, the boxes were covered by a nylon-gauze finely. When the BPH susceptible varieties or TN1 plants were completely dead, the BPH resistance was evaluated by the six-scale scoring system: 0 = no damage; 1 = very slight damage; 3 = 1st and 2nd leaves of most plants partially yellowing; 5 = pronounced yellowing and stunting or about 10–25% of plants wilting; 7 = more than half of the plants wilting or dead and remaining plants severely stunted or dying; 9 = all plants died. The lower score indicates higher resistance, nevertheless, indicating more susceptible to BPH. The evaluation experiments were repeated three times.

### Two-host choice test

A modified protocol was implemented following by Qiu et al., 2010) in host selection behavior experiment. Two 14-days-old seedlings (one pyramided plant with one NIL) with same phenotype were sown at opposite ends of roughly perpendicular diagonals in plastic cup (8 cm-diameter, 15 cm-height). Twenty biological replicates were set for each paired group. The soil of such cups was covered by 2 cm-thichness water layer before infesting. At the four-leaf stage, twenty second to third-instar nymphs were released into such cups that completely covered by a nalon-mesh permitting light and air transmitting. The cups were placed in the greenhouse for preventing the artificial disturbance of human-beings. The number of BPH settled on each plant was counted at 3, 6, 18, 24, 48, 72, 96, 120 h, 144 h after releasing for determining the two-host selection number of BPH, respectively.

### Survival rate of BPH after feeding

Antibiosis always results in the reduction in insect survival, growth rate or reproduction after ingestion of host tissue (Qiu et al., 2011). To check the insect survival rate after BPH feeding, Seeds of LuoYang69, 2 NILs and 93–11 were grown in the plastic cup under natural condition. At the third-leaf stage, each cup/plant was infested with 10 s to third-instar nymphs and the number of surviving insects was counted from 1 to 9 days after BPH release for 9 days. Sixteen biological replicates were set for the assay. It was noticeable that the position that the cup placed should be same. Then, the number of survived BPH at each check point were divided by 10 total BPH released for the BPH survival rate (%).

### Weight gained and honeydew excreted of BPH after feeding

Both the Weight gained of insects (direct) and honeydew excreted by BPH (indirect) after feeding were the important indicators of BPH development. For BPH weight gain and honeydew excretion assay, newly emerged female adults were weighed and enclosed in a pre-weighed Parafilm sachet (each Parafilm sachet contains one female adult) and attached to the leaf sheath of the rice plant. The insects were removed carefully from the sachets after 48 h. Both the insects and each sachet were weighed again. The weight difference of BPH insect was recorded as weight gain of BPH, the weight difference of the Parafilm sachet was recorded as honeydew excretion. These two indicators were measured for 21 biological replicates of the pyramided line, two NILs and the recurrent parent 93–11.

### Test of yield-related agronomic traits and rice grain quality

Ten agronomic traits: plant height, number of panicles per plant, mean length of panicles, number of grains per panicle, number of filled grains per panicle, number of grains per plant, number of filled grains per plant, number of empty glumes per plant, seed setting rate, and 1000-grain weight related tightly with yield of LuoYang69 and 93–11 were investigated in Hubei Academy of Agricultural Sciences under natural field conditions. LuoYang69 and 93–11 individuals were planted in ten rows with row spacing at 30 cm and plant spacing at 20 cm. Twelve individual plants without BPH damage from middle central rows were selected as biological replicates for measurement.

Mature seeds of LuoYang69 and 9311 were harvested in September and were allowed to dry naturally at room temperature. The following rice grain quality traits were tested by dehulled seeds of LuoYang69 and 93–11: brown rice ratio (BR, grains with the inedible outer hull removed), head rice ratio (HR, unbroken and broken translucent grains with at least 3/4 of a whole grain), chalky rice ratio (CR, grain with an opaque, chalky appearance covering half or more of the body of the grain), chalkiness degree (CD), amylose content (AC), alkali spreading value (ASV), grain length (GL), and ratio of grain length to width (L/W). The assessment criterion of rice grain quality followed the national standard GB/T 17891–1999 and NY/T 593–2013. The test of rice grain quality was performed at Institute of Food Crops, Hubei Academy of Agricultural Sciences (Wuhan, China).

### Data analysis

The Chi-square test for goodness-of-fit was done by MS-Excel (Microsoft) and the resistance data and the investigation of agronomic traits were analyzed using statistical one-way ANOVA and comparing the LSD test at a 5% significance level and 1% extremely significance level.

## Results

### Development of NIL*-Bph9* in 93–11 genetic background using MAS

93–11, an elite restorer parent for hybrids, is famous for its good quality, high yield and wide culturing in China. Unfortunately, such elite paddy line was highly susceptible to BPH. To improve BPH resistance of 93–11, 93–11 was used as the recurrent parent to backcross with Pokkali (IRGC 108921, harboring BPH resistance gene *Bph9*) for seven generations and then self-crossed to produce the population of BC_7_F_2_ (Additional file 1: Figure S1) Two flanking markers InD2 and RM28466 tightly linked to *Bph9* locus were used to select the positive progenies for continuous backcrossing. Finally, five lines homozygous at *Bph9* locus were selected as candidate NILs from BC_7_F_2_ populations. A total of 119 polymorphic molecular markers evenly distributed on 12 chromosomes were used to examine the recovery rate of genetic background (RRGB) of candidate NILs. These candidate NILs showed 94.78% to 97.39% genetic identity to 93–11 (Table 1). Then the best individual with the least amount of genetic background noise was selected as NIL*-Bph9* and used to produce BC_7_F_2:3_ populations. These data indicated that *Bph9* has been successfully introgressed into 93–11 through MAS. We further characterized BPH resistance and agronomic traits of NIL-*Bph9*. The results demonstrated that NIL*-Bph9* confers broad-spectrum resistance to BPH without affecting the agronomic performance of the rice plant (Zhao et al., 2016).

**Table 1:** BPH-resistance scores and 93–11 background recovery rate of NIL-*Bph6*, NIL-*Bph9* and pyramided line LuoYang69

	Material No.	Mean Resistance Score	RRGB
Near-isogenic Lines of *Bph6* (BC_5_F_2_)	5Q1100–5–9-11	2.57 ± 0.29^a^	97.72%
Near-isogenic Lines of *Bph9* (BC_7_F_2_)	7Q09–1-9	2.33 ± 0.24^a^	97.39%
	7Q09–2-11	2.44 ± 0.35^a^	96.55%
	7Q09–3-3	2.39 ± 0.20^a^	95.69%
	7Q09–4-5	2.57 ± 0.19^a^	96.58%
	7Q11–1-1	2.59 ± 0.37^a^	94.78%
Pyramided Lines of *Bph9* and *Bph6*	WY17–1-1(LuoYang69)	1.05 ± 0.21^b^	93.18%
	WY18–8-1	1.13 ± 0.18^b^	90.91%
Donor Parent & Recurrent Parents	Pokkali	2.37 ± 0.22^a^	NA
	Swarnalata	2.52 ± 0.25^a^	NA
	93–11	8.17 ± 0.97^c^	100%

Different characters of superscripts (a, b and c) indicate significant differences by one-way ANOVA analysis

### Pyramiding of *Bph6* and *Bph9* in 93–11 genetic background

**Fig. 1:**
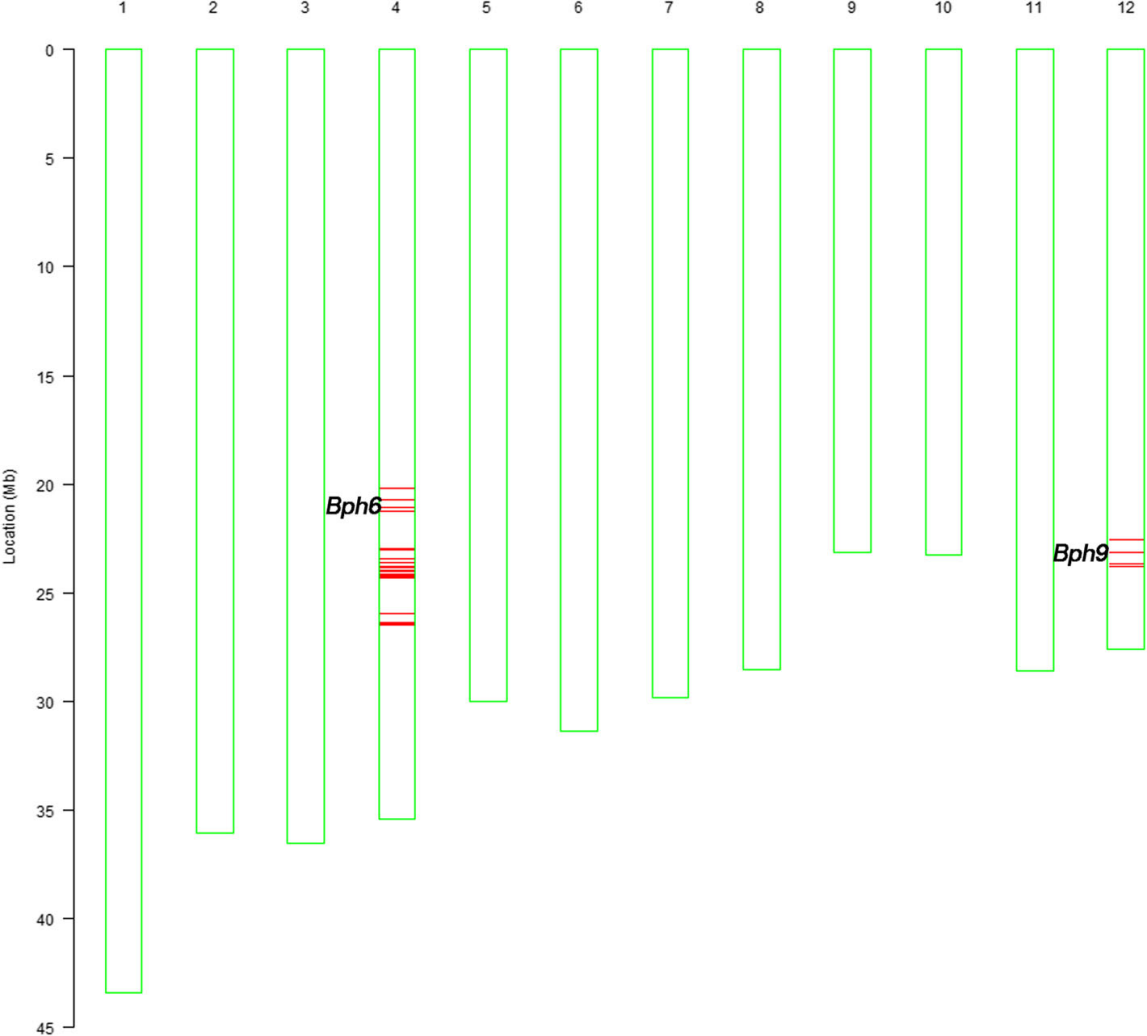
Genetic background assay of LuoYang69 using the RICE6K array. The red lines on chromosome 4 and chromosome 12 indicate the SNP loci with homozygous genotypes where genomic fragments of the donor parent Swarnalata (*Bph6*) and Pokkali (*Bph9*) were introgressed, respectively

It has been proposed that some varieties bearing single BPH resistance gene were quickly broken down within a few years due to rapid adaptation of BPH (Khush, 1977). Pyramiding multiple BPH resistance genes is an efficient strategy to develop more durable resistant varieties against BPH. The rice variety Swarnalata contained BPH resistance gene *Bph6* and showed a high level of resistance to BPH biotypes 4 and mixed BPH biotypes in China. Previously, we fine-mapped *Bph6* and developed NIL-*Bph6* in 93–11 genetic background (Qiu et al., 2010). To pyramid *Bph6* and *Bph9* and develop more durable BPH resistance rice, NIL-*Bph6* and NIL-*Bph9* were crossed in this study. Two pyramided lines with both homozygous *Bph6* and *Bph9* were selected by marker H and InD2 from F_2_ plants (Additional file 2: Figure S2). The genetic background of these two selected pyramided lineswas assayed using evenly distributed 95 polymorphic markers among the Swarnalata, Pokkali and 93–11 (Table 1). The genetic background of WY17–1-1 was further analyzed by more powerful RICE6K SNP array. The result showed that WY17–1-1 almost had the same genetic background as 93–11, except the locus of *Bph6* and *Bph9* introgressed from genetic background of Swarnalata and Pokkali (Fig. 1). The introgressed genomic fragments containing *Bph6* and *Bph9* are about 6.3 Mb (from 20,187,393 to 26,503,057 bp on chromosome 4) and 1.2 Mb (from 22,532,464 to 23,776,103 bp on chromosome 12), respectively (Fig. 1). WY17–1-1 was then designated as LuoYang69 (for pyramiding of *Bph6* and *Bph9*) and subjected to subsequent BPH-resistance evaluation and agronomic traits investigation (Table 1).

### Evaluation of BPH resistance of LuoYang69

**Fig. 2:**
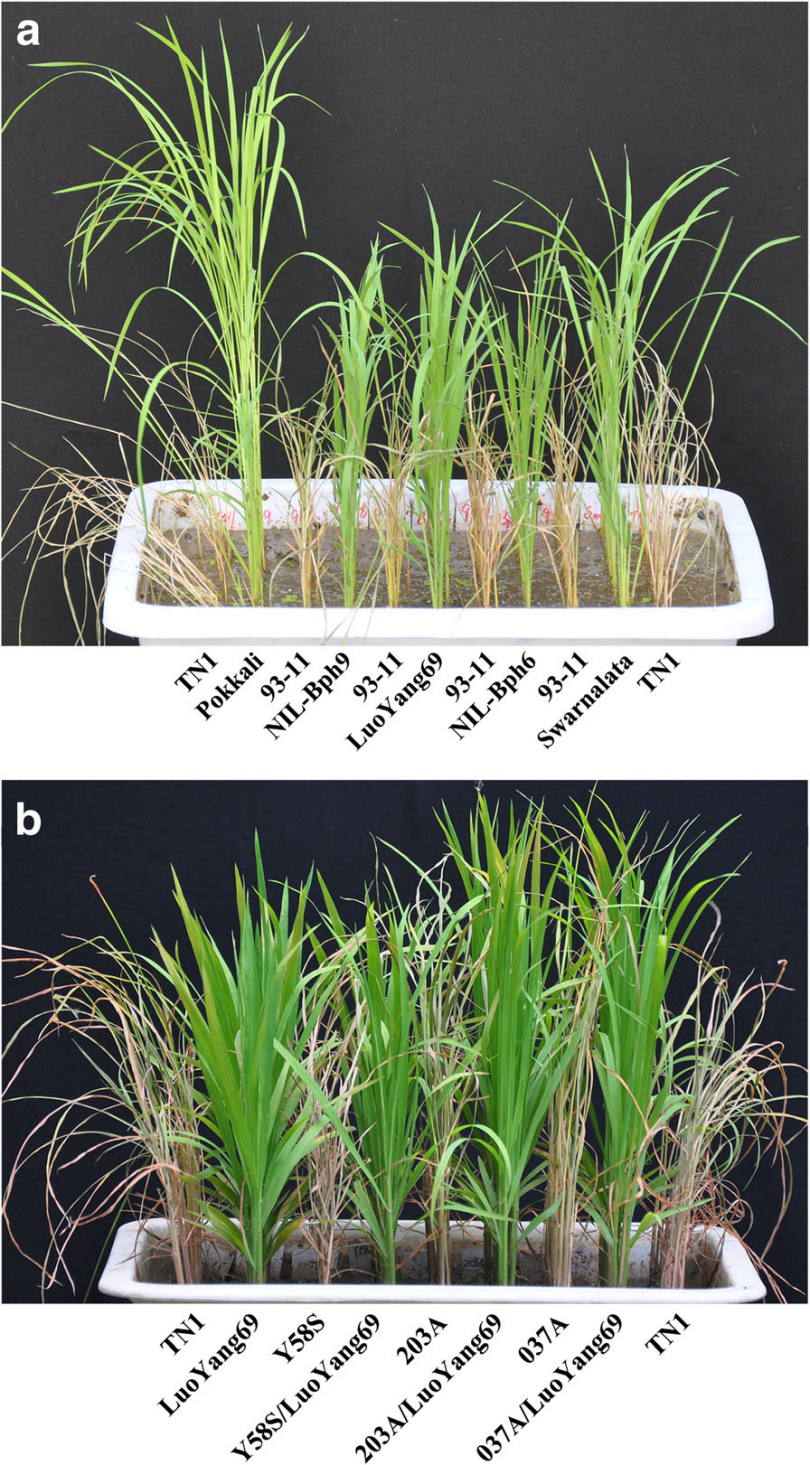
BPH resistance assay of LuoYang69. **a** BPH resistance assay of near-isogenic lines and pyramided line. **b** BPH resistance assay of hybirds and pyramided lines. LuoYang69, *Bph6* and *Bph9* pyramided line in 93-11 genetic background; Pokkali and Swarnalata are the resistance donors of *Bph9* and *Bph6*,respectively; NIL-*Bph6* and NIL-*Bph9* are the near-isogenic lines harboring *Bph6* and *Bph9* in 93-11 genetic background, respectively; Y58S, TGMS line; Y58S/LuoYang69, hybrid derived from Y58S and LuoYang69; 037A and 203A, HL type CMS lines; 037A/LuoYang69 and 203A/LuoYang69, hybrids derived from 037A and LuoYang69, 203A and LuoYang69, respectively; TN1, BPH-susceptible rice variety

To test whether LuoYang69 could improve the BPH resistance than either NIL-*Bph6* or NIL-*Bph9*, firstly we evaluated the BPH resistance of these lines at the seedling stage under greenhouse conditions. While the recurrent parent 93–11 and susceptible control TN1 died completely, LuoYang69, along with NIL-*Bph6* and NIL-*Bph9*, showed high levels of resistance to BPH (Fig. 2a). The BPH resistance scores of NIL-*Bph6* and NIL-*Bph9* were 2.57 ± 0.29 and 2.33 ± 0.24, respectively. Compared with either of NIL-*Bph6* and NIL-*Bph9*, *Bph6* and *Bph9* pyramided line LuoYang69 conferred significantly enhanced resistance to BPH with resistance score of 1.05 ± 0.12 (*P* < 0.01), reflecting the additive effect of resistance gene pyramiding (Table 1).

To explore the potential usefulness of LuoYang69 for breeding BPH resistant hybrids,

LuoYang69 was crossed with TGMS line Y58S, HL type CMS lines 037A and 203A, and the derived hybrids were evaluated the BPH resistance. The hybrids Y58S/LuoYang69, 203A/LuoYang69 and 037A/LuoYang69 were highly resistant to BPH, with the resistance scores of 1.48, 1.24 and 1.43, respectively (Fig. 2b). More importantly, no significant differences of resistance scores were observed between such hybrids and LuoYang69. Taken together, the results demonstrated that both *Bph6* and *Bph9* are complete dominance genes and LuoYang69 derived hybrids with heterozygous *Bph6* and *Bph9* genes could also confer high level of resistance to BPH.

**Fig. 3:**
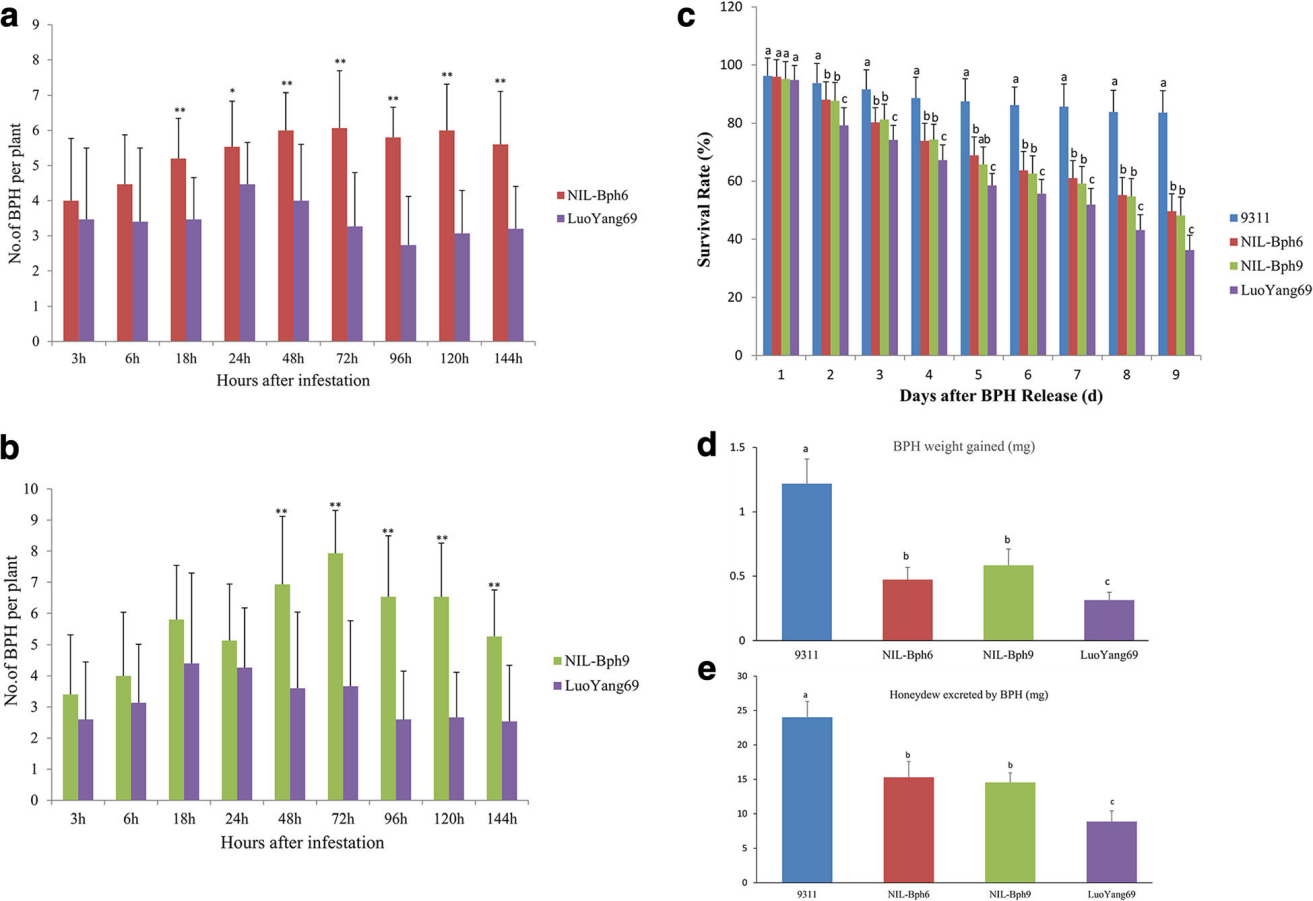
Antixenosis and antibiosis effects of LuoYang69 on BPH insects. **a** Settling of BPH insects on LuoYang69 and on NIL-*Bph6* in a two-host choice test. **b** Settling of BPH insects on LuoYang69 and on NIL-*Bph9* in a two-host choice test. **c** BPH survival rate on LuoYang69, *NIL-Bph6*, *NIL-Bph9* and 93–11 in a non-choice feeding experiment, respectively. **d** Weight gain of BPH insects after feeding on LuoYang69, *NIL-Bph6*, *NIL-Bph9* and 93–11 for 48 h, respectively. **e** Measurement of honeydew excreted from BPH insects on LuoYang69, *NIL-Bph6*, *NIL-Bph9* and 93–11, respectively. **a** and **b** Bars represent means of 20 replicates. Error bars represent the SD. Means labeled with asterisks are significantly different (*P* < 0.05). Means labeled with double asterisks are extremely significant different (*P* < 0.01). **c** Bars represent means of 16 replicates. Error bars represent the SD. The different letters above the bars are significantly different at *P* < 0.05 level. **d** and **e** Bars represent means of 21 replicates. Error bars represent the SD. The different letters above the bars are significantly different at *P* < 0.05 level

Generally, rice plants have evolved two major resistance strategies against herbivores: antixenosis that affects insect settling, colonization, or oviposition and antibiosis that reduces insect feeding, growth, or survival **(**Qiu et al., 2011 **)**. In two-host choice tests, many more BPH insects preferred to settle on NIL-*Bph6* or NIL-*Bph9* than on LuoYang69 plants, respectively. Noticeably, the number of settled BPHs on LuoYang69 was constant low (<4 mostly) at all time-points checked. Compared with NIL-*Bph6*, the numbers of BPH settled on LuoYang69 decreased significantly from the 18 h to 144 h(P < 0.01)after BPH release (Fig. 3a). Compared with NIL-*Bph9*, the numbers of BPH settled on Luoyang69 plants declined significantly 48 h after BPH release over the study period (P < 0.01) (Fig. 3b). The results above indicated that LuoYang69 has a much stronger antixenotic effect on BPH than either of NIL-*Bph6* and NIL-*Bph9.*

To test whether LuoYang69 improves the antibiosis effect against BPH than NIL-*Bph6* or NIL-*Bph9*, BPH survival rate was investigated on LuoYang69, *NIL-Bph6*, *NIL-Bph9* and 93–11, respectively. As shown in Fig. 3c, no significant differences of BPH survival rate were observed among these plants at the first day of BPH infestation. While the average numbers of surviving BPHs on LuoYang69, *NIL-Bph6* and *NIL-Bph9* decreased gradually and showed a significant difference in numbers compared with 93–11 from the second day to the last day of BPH infestation. Remarkably, the average numbers of surviving BPHs on LuoYang69 also showed a significant difference compared with either *NIL-Bph6* or *NIL-Bph9* from the second day of BPH infestation. At the end of investigation, the BPH survival rates of LuoYang69, *NIL-Bph6* and *NIL-Bph9* dropped to 35.7%, 49.3% and 48.5%, respectively (F = 14.791, *P* = 0.003 compared by *NIL-Bph9* with Luoyang69; F = 11.37, *P* = 0.004 compared by *NIL-Bph6* with Luoyang69 (Fig. 3c). These findings suggest *Bph6* and *Bph9* pyramided line LuoYang69 had stronger antibiosis effect on BPH than NIL-*Bph6* and NIL-*Bph9*.

To determine whether LuoYang69 affects BPH growth and development more greatly than NIL-*Bph6* or NIL-*Bph9*, we compared honeydew excretion and weight gain of BPH feeding on these plants. The results indicated that BPH insects fed on LuoYang69 plants showed significantly lower weight gain and honeydew excretion than those on NIL-*Bph6* or NIL-*Bph9* (Fig. 3d-e). Taken together, these results demonstrate that *Bph6* and *Bph9* pyramided line LuoYang69 has a much stronger antixenotic and antibiosis effect on BPH than NIL-*Bph6* and NIL-*Bph9*, which contributes to its significantly enhanced resistance against BPH.

### The performance of agronomic traits and rice grain quality of LuoYang69

Unexpected linkage drag is a common phenomenon in disease and insect resistance breeding programs, which affects yield and grain quality characteristics of rice cultivars (Yeo and Shon, 2001; Liu et al., 2009). To test whether the agronomic traits and rice grain quality of *Bph6* and *Bph9* pyramided line LuoYang69 were identical to its recurrent parent 93–11 under normal growth conditions, we evaluated ten agronomic traits of LuoYang69 and 93–11 in Wuhan nearly without BPH damage. Most of agronomic traits such as plant height, panicle number, panicle length, number of filled grains per panicle, total grain number, seed setting rate of LuoYang69 showed no significant difference compared with 93–11. While the 1000-grain weight of LuoYang69 was about 0.54 g heavier than that of 93–11 (*P* < 0.05) (Table 2). Moreover, most of the rice grain quality traits (brown rice ratio, head rice ratio, alkali spreading value, amylose content, grain length, grain width, ratio of grain length to width) of LuoYang69 were nearly identical to its recurrent parent 93–11. While Luoyang69 had better rice quality with a lower chalky rice ratio and chalkiness degree compared with 93–11 (Table 3). These results indicated that pyramiding of *Bph6* and *Bph9* in 93–11 genetic background did not affect the elite agronomic traits and rice grain quality of 93–11. The resulting LuoYang69 will be valuable resource to develop hybrid rice with high BPH resistance and excellent agronomic performance.

**Table 2:** The agronomic traits of LuoYang69 and 93–11

	Plant Height (cM)	Panicle Number	Panicle Length (cM)	Number of Grains per Panicle	Number of Filled Grains per Panicle	Number of Grains per Plant	Number of Filled Grains per plant	Number of Empty Glume per plant	Seed setting rate (%)	*1000-Grain Weight (g)
93–11	128.93 ± 2.12	6.73 ± 0.59	23.47 ± 0.42	188.41 ± 13.25	153.02 ± 12.39	1268.67 ± 149.13	1031.2 ± 136.22	237.47 ± 30.26	81.19 ± 2.25	30.43 ± 0.34
LuoYang69	129.79 ± 1.67	6.67 ± 0.98	23.11 ± 0.77	178.89 ± 14.99	146.09 ± 11.89	1182.20 ± 120.10	966.47 ± 108.55	215.73 ± 30.25	81.71 ± 2.27	30.97 ± 0.48

Labeled asterisk indicated significantly different (*P* < 0.05)

**Table 3:** The rice quality traits of LuoYang69 and 93–11

	Brown Rice ratio (%)	Head Rice ratio (%)	Grain Length (mm)	Ratio of Grain Length to Width	Chalky Rice ratio (%)	Chalkiness Degree (%)	Amylose Content (%)	Alkali Spreading value
93–11	76.2	68.1	6.5	3.1	33	9.2	13.0	6.0
LuoYang69	75.2	66.6	6.6	3.1	26	6.0	11.7	6.0

### Development and validation of functional marker for *Bph9*

**Fig. 4:**
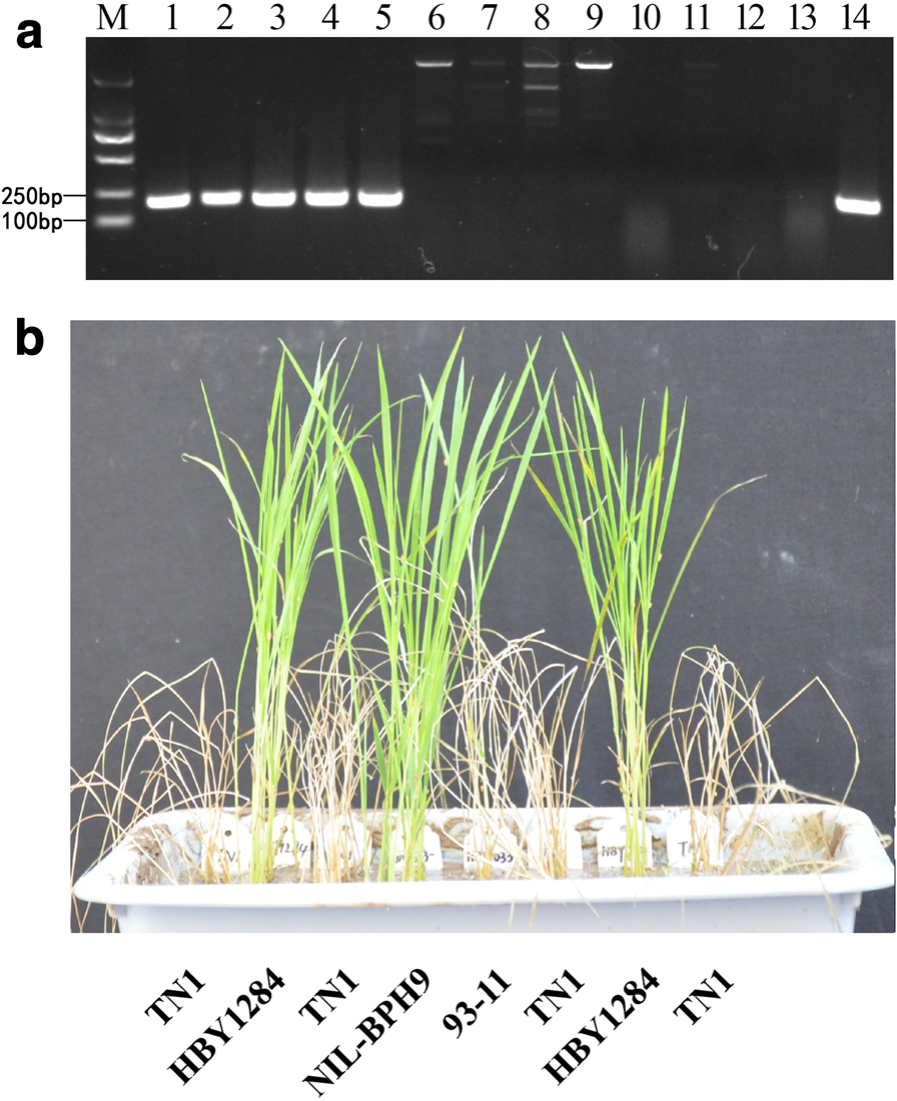
Development and validation of functional dominant marker for *Bph9*. **a** PCR products of genomic DNA from rice cultivars using *Bph9*-specific dominant marker TPT202/TR3. M, DL2000; 1 to 14: Pokkali, HBY1284, BM 13, KAU 1734–2, CR 157–392-284, ASD7, Mudgo, T12, IR54742, IR65482–7–216-1-2, IR71033–121–15, ADR52, 93–11, LuoYang69. The size of PCR product was 195 bp. **b** BPH resistance assay of HBY1284 identified by *Bph9*-specific dominant marker

In a recent study, we isolated *Bph9* and revealed that *Bph9* encodes a rare type of nucleotide-binding and leucine-rich repeat (NLR) gene (Zhao et al., 2016). To develop PCR-based functional molecular marker for *Bph9*, we first compared the genomic sequences of *Bph9* alleles in rice varieties Pokalli, 93–11 and Nipponbare. The result showed that a 1.2 kb specific fragment was inserted in intron 1 of *Bph9* allele in Pokalli, absent in the corresponding regions of *Bph9* alleles in Nipponbare and 93–11. To detect the insertion in intron 1 of *Bph9* allele in Pokalli, we designed one forward primer in 3′ sequence of the insertion and one reverse primer spanning the insertion and its flanking sequence. Using this marker, we genotyped the insertion in Pokalli and 20 susceptible cultivars, as well as the original donors of other seven BPH resistance genes (*Bph1*, *bph2*, *bph7*, *Bph10*, *Bph18*, *Bph21*, and *Bph26*) clustered on the long arm of chromosome 12 and actually allelic with each other (Zhao et al., 2016). As shown in Fig. 4a, a 195-bp fragment of the insertion was amplified by B9D only from Pokalli, while no amplification was obtained from the other cultivars, including those resistant allelotypes of *Bph9*. B9D might be a dominant *Bph9*-specific marker which can be used for detecting the presence or absence of *Bph9* in rice varieties. As the dominant marker could not differentiate the homozygous and heterozygous status of the alleles, which would limit its application in molecular breeding program. Thus, we developed a codominant InDel marker IR2 according to sequence comparison of *Bph9* with its alleles from rice varieties and landraces. Though IR2 itself could not distinguish *Bph9* and *Bph18* (data not shown), the combination of B9D and IR2 could facilitate MAS of *Bph9* in BPH resistance breeding programs.

We further genotyped rice germplasm to investigate distribution of *Bph9* using this *Bph9*-specific dominant marker. The genotyping analysis indicated 195-bp specific fragment was amplified in 8 cultivars of 673 rice varieties assayed successively (Additional file 3: Table S1), including HBY1284 (WD15610) from 299 accessions of the mini core collection of rice germplasm provided by Hubei Academy of Agricultural Science; BM13(WD14400), KAU1734–2 (WD15283) and CR157–392-284(WD17628) from 89 resistant paddy materials provided by China National Rice Research Institute; MGL2 (IRGC 6218), CHERIVIRUPPU (IRGC 19928), POKKALI 8558 (IRGC 26869) and CR157–392-4 (IRGC 39247) from 285 resistant paddy materials of IRRI provided by Huazhong Agricultural University. All of these 8 cultivars were resistant to BPH (Table 4), as exemplified by HBY1284 (Fig. 4b). The genomic sequences and full-length cDNA of the gene corresponding to *Bph9* were further obtained from rice varieties HBY1284, WD14400, WD15283 and WD17628. The sequence comparison revealed that *Bph9* alleles from these 4 cultivars share 100% sequence identity with *Bph9* in Pokkali (IRGC 108921). Thus, we did not determine genomic sequences of *Bph9* alleles from newly identified *Bph9* harboring rice varieties MGL2 (IRGC 6218), CHERIVIRUPPU (IRGC 19928), POKKALI 8558 (IRGC 26869) and CR157–392-4 (IRGC 39247). These results validated that B9D is a dominant *Bph9*-specific marker which can effectively distinguish rice varieties harboring *Bph9.*

**Table 4:** Rice cultivars harboring *Bph9* identified by B9D

Accession No.	Accession Name	Origin	Resistance gene	Resistance score	Sequence Identical to *Bph9*
WD-14400	BM 13	Philippines	*Bph9*	3.12 ± 0.29	√
WD-15283	KAU 1734–2	India	*Bph9*	3.54 ± 0.35	√
WD-17628	CR 157–392-284	India	*Bph9*	2.97 ± 0.26	√
WD-15610	HBY1284	India	*Bph9*	2.87 ± 0.22	√
IRGC 6218	MGL 2	India	*Bph9*	3.01 ± 0.27	ND
IRGC 19928	CHERIVIRUPPU	India	*Bph9*	4.69 ± 0.35	ND
IRGC 39247	CR157–392-4	India	*Bph9*	2.77 ± 0.24	ND
IRGC 26869	POKKALI(8558)	Sri Lanka	*Bph9*	4.83 ± 0.33	ND

## Discussion

The brown planthopper (BPH) is the most damaging pest of rice (*Oryza sativa*.L) and a serious threat to rice production in Asia (Liu et al., 2015). Host-plant resistance has been proposed as the most economic effective and environmental-friendly approach to reduce BPH damage and increase yield potential of cultivars. The resistance is mainly controlled by major genes and now at least 31 BPH resistance genes have been identified genetically in the cultivated rice germplasm and wild *Oryza* species sources. Thirteen BPH resistance genes have been cloned via map-based cloning approach (Ji et al., 2016; Ren et al., 2016; Du et al., 2009; Tamura et al., 2014; Wang et al., 2015; Zhao et al., 2016; Liu et al. 2015; Jing et al., 2017). These identified BPH resistance genes provided a solid foundation for marker assisted selection (MAS) in breeding programs. However, the varieties bearing single BPH resistance gene were quickly broken down within a few years due to rapid adaptation of BPH or evolution of new biotypes (Jena and Kim, 2010). Resistant varieties with single resistance gene *Bph1* were released in 1973 and saved rice production from massive BPH damage. However, their resistance was broken down in 1976 with the development of a new BPH population (biotype 2). Varieties with *bph2* showing effective resistance were then released and widely grown, but again, these varieties were also adapted by another BPH population (biotype 3). Several studies showed some highly and durable resistant varieties carry one or more major genes, along with minor QTLs contributing to BPH resistance. For example, *Bph1* carrying rice variety IR64 showed durable and stable resistance after the spread of *Bph1*-breaking BPH populations (biotype 2), which was conferred by seven minor QTLs detected on chromosomes 1, 2, 3, 4, 6 and 8 (Alam and Cohen, 1998a). Indica cultivar ADR52, highly resistant to BPH, contained two major genes *Bph25* and *Bph26* along with several associated minor QTLs (Myint et al., 2012, Srinivasan et al., 2015). Progress has been made in pyramiding major BPH resistance genes into elite cultivars. Pyramiding of *Bph6* and *Bph12* resulted in a higher resistance level than that of near isogenic lines of single gene, showing an additive effect against BPH (Qiu et al., 2012). *Bph3* and *Bph27(t)* pyramided line displayed higher resistance than those lines with single resistance gene (Liu et al., 2016). *Bph14* and *Bph15* also had a strong dosage effect on the resistance to BPH, are widely used in BPH resistance gene pyramiding breeding practice (Li et al., 2006; Xia et al., 2010; Zhu et al., 2013a; Zhu et al., 2013b; Hu et al., 2012; Hu et al., 2013; Hu et al., 2015; Cai et al., 2015; Wang et al., 2016). These studies demonstrated that pyramiding multiple BPH resistance genes is an efficient strategy to develop higher and more durable resistant varieties against BPH.

*Bph6*, originally identified in Swarnalata, conferred high level of resistance to BPH biotype 4 (Bangladesh BPH population) and BPH population in China (biotype 2 being the dominant one). *Bph6* was fine-mapped on the long arm of chromosome 4 (Qiu et al., 2010). *Bph9* in Pokkali was also a high and broad-spectrum BPH- resistance gene, encoding a rare type of nucleotide-binding and leucine-rich repeat (NLR) gene (Zhao et al., 2016). *Bph6* and *Bph9*, which were both explored from the traditional tropical *indica* cultivars, could be the more adopted genes in rice BPH-resistance pyramiding breeding programs for less linkage drag of unbeneficial traits than those from the wild species. In the present study, we introgressed *Bph9* into elite cultivar 93–11 through MAS. Then, *Bph6* and *Bph9* were pyramided into 93–11 by intercrossing with near isogenic lines NIL-*Bph6* and NIL-*Bph9*. Compared with either of NIL-*Bph6* and NIL-*Bph9*, the resulting *Bph6* and *Bph9* pyramided line LuoYang69 conferred significantly enhanced resistance to BPH with resistance score of 1.05 ± 0.12 (*P* < 0.01), reflecting the additive effect of resistance gene pyramiding. The further detailed analyses indicated that *Bph6* and *Bph9* pyramided line LuoYang69 had a much stronger antixenotic and antibiosis effects on BPH than those near isogeniclines with single resistance gene. The evaluation of agronomic traits and rice grain quality of LuoYang69 showed that most of traits were identical to its recurrent parent 93–11. Noticeably, the 1000-grain weight of LuoYang69 was about 0.54 g heavier than that of 93–11 (*P* < 0.05). These results demonstrated that *Bph6* and *Bph9* pyramided line LuoYang69 developed in this study would be valuable resource to develop hybrid rice with high BPH resistance and excellent agronomic performance.

In BPH resistance breeding programs, it is important to make clear whether resistance genes can be used for hybrid improvement and production by checking effects of resistance genes in the homozygous and heterozygous state. When pyramiding *Bph14* and *Bph15* into rice restorer lines and hybrids, Wang et al., 2016) found that hybrids carrying both homozygous resistance genes showed stronger resistance to BPH. However, the hybrids with heterozygous *Bph14* and *Bph15* remained susceptible to BPH. Hu et al., 2013) found that the hybrids containing *Bph14* and *Bph18* in a homozygous state, had moderate resistance to BPH. However, the hybrids harboring such both resistance genes in a heterozygous state, were susceptible to BPH. In this study, LuoYang69 derived hybrids containing heterozygous *Bph6* and *Bph9* genes also conferred high level of resistance to BPH, similar to that of LuoYang69 with the homogenous genotypes of *Bph6* and *Bph9*. This demonstrated that both *Bph6* and *Bph9* are complete dominance genes and combining such both genes would be a practical strategy of hybrid improvement and production for rice breeders.

Molecular markers tightly linked to the identified BPH resistance genes are playing the important role in MAS based gene pyramiding breeding programs for the rapid selection of genotypes with targeted gene (Collard et al., 2005; Hayashi et al., 2010). However, the use of such tightly linked markers bears the risk of being lost through genetic recombination between the marker and the target gene, resulting in misdiagnosis of traits of interest. Such problem can be overcome by the application of functional markers (FMs) derived from DNA polymorphisms found in allelic variants of a functional gene. FMs developed from cloned resistance genes are being used in MAS-based breeding (Fjellstrom et al., 2004; Perumalsamy et al., 2010). The combination of markers *Bph14P*/*Bph14N* targeting promotor and LRR region of BPH resistance gene *Bph14*, respectively, were verified in two breeding populations and a Chinese mini core collection of *Oryza sativa* (Zhou et al., 2013). Our recent work revealed that the eight BPH resistance genes clustered on the long arm of chromosome 12 *(Bph1*, *bph2*, *bph7*, *Bph9*, *Bph10*, *Bph18*, *Bph21*, *Bph26*) are actually allelic with each other and can be divided into four allelotypes. We analyzed nucleotide variation of *Bph9* alleles from rice varieties and landraces. According to sequence comparison of *Bph9* with its alleles, we developed and validated a *Bph9*-specific functional dominant marker B9D, which can easily distinguish *Bph9* with other cultivars, including its resistant allelotypes *(Bph1*, *bph2*, *bph7*, *Bph10*, *Bph18*, *Bph21*, *Bph26*). Using *Bph9*-specific functional dominant marker, we screened and evaluated worldwide accessions of rice germplasm. Of the 673 varieties tested, eight were identified to harbor *Bph9* and showed resistance to BPH. The sequences of *Bph9* alleles in these cultivars were identical to that of *Bph9* in Pokkali (IRGC 108921), suggesting that the functional *Bph9* locus is derived from a common progenitor. The results also demonstrated that *Bph9*-specific functional dominant marker B9D is a reliable marker to isolate cultivars harboring the functional *Bph9* gene from rice germplasm worldwide. These identified *Bph9* containing cultivars can be used as new sources for BPH resistance breeding programs. To differentiate the homozygous and heterozygous status of *Bph9*, the present work also developed a codominant InDel marker IR2. The combination of B9D and IR2 could either validate the existence of *Bph9* or indicate the diploid genotype of paddy lines, which will facilitate MAS of *Bph9* in BPH resistance breeding programs.

## Conclusion

We pyramided *Bph6* and *Bph9* into elite restorer line 93–11 through MAS. The resulting *Bph6* and *Bph9* pyramided line LuoYang69 had stronger antixenotic and antibiosis effects on BPH and exhibited significantly enhanced resistance than near isogenic lines NIL-*Bph6* and NIL-*Bph9*. Its derived hybrids harboring heterozygous *Bph6* and *Bph9* genes also conferred high level of resistance to BPH. The development of LuoYang69 pyramided with *Bph6* and *Bph9* provides valuable resource to develop hybrid rice with high BPH resistance and excellent agronomic performance. We also developed functional markers for MAS of *Bph9* in rice breeding programs. The 8 identified *Bph9* harboring cultivars can be used as new sources BPH resistance breeding programs.

## Additional files

Additional file 1: Figure S1. Strategy used to pyramid *Bph6* and *Bph9* in 93–11 genetic background. Pokkali and Swarnalata are the resistance donors of *Bph9* and *Bph6*, respectively. The resulting *Bph6* and *Bph9* pyramided line in 93–11 genetic background is designated as LuoYang69. (TIFF 330 kb)

Additional file 2: Figure S2. PCR analysis of LuoYang69 with molecular markers InD2 (for *Bph9*) and H (for *Bph6*). M: DL5000; 1 to 6: 93–11, Swarnalata, NIL-*Bph6*, Pokkali, NIL-*Bph9*, LuoYang69*. (TIFF 2603 kb)*

Additional file 3: Table S1. Rice germplasm resources genotyped with *Bph9*-specific dominant marker. Labeled asterisk indicated cultivars haboring *Bph9*. (XLSX 16142 kb)

## Abbreviations

AC: Amylose content
ASV: Alkali spreading value
BPH: Brown planthopper
BR: Brown rice ratio
CD: Chalkiness degree
CK: Chalky grain rate
CMS: Cytoplasmic male sterility
CTAB: Hexadecyl trimethyl ammonium bromide
FM: Functional marker
HR: Head rice rate
Indel: Insertion-deletion
IRRI: International Rice Research Institute
L/W: Ratio of grain length to width
LSD: Least significant difference tests
MAB: Molecular marker-assisted backcross
MAS: Marker-assisted selection
NIL: Near isogenic line
NLR: Nucleotide-binding and leucine-rich repeat
PCR: Polymerase chain reaction
RAPD: Random amplified polymorphic DNA
RFLP: Restriction fragment Length polymorphism
RGSV: Rice grassy stunt virus
RRGB: Recovery rate of genetic background
RRSV: Rice ragged stunt virus
RWSV: Rice wilted stunt virus
SNP: Single nucleotide polymorphism
SSR: Simple sequence repeat
STS: Sequence-tagged sites
TE: Tris and EDTA buffer
TGMS: Thermo-sensitive genetic male-sterile lines

## References

[CR1] Alam SN, Cohen MB (1998). Detection and analysis of QTLs for resistance to the brown planthopper, *Nilaparvata lugens*, in a doubled-haploid rice population. Theor Appl Genet.

[CR2] Alam SN, Cohen MB (1998). Durability of brown planthopper, *Nilaparvata lugens*, resistance in rice variety IR64 in greenhouse selection studies. Entomol Exp Appl.

[CR3] Brar DS, Virk PS, Jena KK, Khush GS (2009) “Breeding for resistance planthoppers in rice”. In Planthoppers: new threats to the sustainability of intensive rice production systems in Asia, Edited by: Heong KL and Hardy B. Los Baños: International Rice Research Institute. p. 401–427

[CR4] Cai Z, Li J, Zhou D, Fu H (2015). Improving the resistance of japonica in the north of Zhejiang province by marker-assisted selection (in Chinese with English abstract). Mod Agric Sci Technol.

[CR5] Collard BCY, Jahufer MZZ, Brouwer JB (2005). An introduction to markers, quantitative trait loci (QTL) mapping and marker-assisted selection for crop improvement: the basic concepts. Euphytica.

[CR6] Du B, Zhang W, Liu B, Hu J, Wei Z, Shi Z, He R, Zhu L, Chen R, Han B, He G (2009). Identification and characterization of *Bph14*, a gene conferring resistance to brown planthopper in rice. Proc Natl Acad Sci U S A.

[CR7] Fjellstrom R, Conaway-Bormans CA, McClung AM, Marchetti MA, Shank AR, Park WD (2004). Development of DNA markers suitable for marker assisted selection of three *Pi* genes conferring resistance to multiple Pyricularia grisea pathotypes. Crop Sci.

[CR8] Hayashi K, Yasuda N, Fujita Y, Koizumi S, Yoshida H (2010). Identification of the blast resistance gene *Pit* in rice cultivars using functional markers. Theor Appl Genet.

[CR9] Heinrichs EA, Moramorosch K, Harris KF (1979). Control of leafhopper and planthopper vectors of rice viruses. Leafhopper vectors and planthopper disease agents.

[CR10] Hu J, Cheng MX, Gao GJ, Zhang QL, Xiao JH, He YQ (2013). Pyramiding and evaluation of three dominant brown planthopper resistance genes in the elite indica rice 9311 and its hybrids. Pest Manag Sci.

[CR11] Hu J, Li X, Wu CJ, Yang CJ, Hua HX, Gao GJ, Xiao JH, He YQ (2012). Pyramiding and evaluation of the brown planthopper resistance genes *Bph14* and *Bph15* in hybrid rice. Mol Breeding.

[CR12] Hu J, Xiao C, He Y (2016). Recent progress on the genetics and molecular breeding of brown planthopper resistance in rice. Rice.

[CR13] Hu W, Li Y, Hu K, Jiang Y, Zhang Y (2015). Improvement BPH-resistance of rice culitivar Guinongzhan by marker-assisted selection for BPH-resistant genes (in Chinese with English abstract). Mol Plant Breed.

[CR14] Huang Z, He GC, Shu LH, Li XH, Zhang QF (2001). Identification and mapping of two brown planthopper resistance genes in rice. Theor Appl Genet.

[CR15] Jena KK, Kim SM (2010). Current status of brown planthopper (BPH) resistance and genetics. Rice.

[CR16] Ji H, Kim SR, Kim YH (2016). Map-based cloning and characterization of the *BPH18* gene from wild Rice conferring resistance to Brown Planthopper (BPH) insect Pest. Sci Reports.

[CR17] Jing SL, Zhao Y, Du B, Chen RZ, Zhu LL, He GC (2017). Genomics of interaction between the brown planthopper and rice. Curr Opin Insect Sci.

[CR18] Khush GS (1977). Breeding for resistance in rice. New York Acad Sci.

[CR19] Lakshmi VJ, Krishnaiah NK, Katti G, Pasalu IC, Bhanu KV (2010). Development of insecticide resistance in rice brown planthopper and whitebacked planthopper in Godavari Delta of Andhra Pradesh. Indian J. Plant Prot.

[CR20] Li J, Xia M, Qi H, He G, Wan B, Zha Z (2006). Marker-assisted selection for brown planthopper (*Nilaparvata lugens* Stål) resistance genes Bph14 and Bph15 in rice. Science Agricultura Sinica.

[CR21] Liu WQ, Fan YY, Chen J, Shi YF, Wu JL (2009). Avoidance of linkage drag between blast resistance gene and the QTL conditioning spikelet fertility based on genotype selection against heading date in Rice. Rice Sci.

[CR22] Liu YL, Chen LM, Liu YQ (2016). Marker assisted pyramiding of two brown planthopper resistance genes, *Bph3* and *Bph27 (t)*, into elite rice cultivars. Rice.

[CR23] Liu YQ, Wu H, Chen H, Liu YL, He J, Kang HY, Sun ZG, Pan G, Wang Q, Hu JL, Zhou F, Zhou KN, Zheng XM, Ren YL, Chen LM, Wang YH, Zhao ZG, Lin QB, Wu FQ, Zhang X, Guo XP, Cheng XN, Jiang L, Wu CY, Wang HY, Wan JM (2015). A gene cluster encoding lectin receptor kinases confers broadspectrum and durable insect resistance in rice. Nat Biotechnol.

[CR24] Matsumura M, Takeuchi H, Satoh M, Heong KL, Hardy B (2009). Current status of insecticide resistance in rice planthoppers in Asia. Planthoppers: new threats to the sustainability of intensive rice production systems in Asia.

[CR25] Matteson PC (2000). Insect pest management in tropical Asian irrigated rice. Annu Rev Entomol.

[CR26] Myint KK, Fujita D, Matsumura M, Sonoda T, Yoshimura A, Yasui H (2012). Mapping and pyramiding of two major genes for resistance to the brown planthopper (*Nilaparvata lugens* [Stål]) in the rice cultivar ADR52. Theor Appl Genet.

[CR27] Nemoto H, Ikeda R, Kaneda C (1989). New genes for resistance to brown planthopper, *Nilaparvata lugens* Stål, in rice. Jpn J Breed.

[CR28] Normile D (2008). Agricultural research. Reinventing rice to feed the world. Science.

[CR29] Park DS, Lee SK, Lee JH (2007). The identification of candidate rice genes that confer resistance to the brown planthopper (*Nilaparvata lugens*) through representational difference analysis. Theor Appl Genet.

[CR30] Pathak MD, Cheng CH, Fortuno ME (1969). Resistance to *Nephotettix impicticeps* and *Nilaparvata lugens* in varieties of rice. Nature.

[CR31] Perumalsamy S, Bharani M, Sudha M, Nagarajan P, Arul L, Saraswathi R, Balasubramanian P, Ramalingam J (2010). Functional marker-assisted selection for bacterial leaf blight resistance genes in rice (*Oryza sativa* L.). Plant Breed.

[CR32] Porebski S, Grant BL, Bernard RB (1997). Modification of CTAB DNA extraction protocol for plants containing high polysaccharide and polyphenol components. Plant Mol Biol Report.

[CR33] Qiu YF, Guo JP, Jing SL, Tang M, Zhu LL, He GC (2011). Identification of antibiosis and tolerance in rice varieties carrying brown planthopper resistance genes. Entomol Exp Appl.

[CR34] Qiu YF, Guo JP, Jing SL, Zhu LL, He GC (2010). High-resolution mapping of the brown planthopper resistance gene Bph6 in rice and characterizing its resistance in the 93-11 and Nipponbare near isogenic backgrounds. Theor Appl Genet.

[CR35] Qiu YF, Guo JP, Jing SL, Zhu LL, He GC (2012). Development and haracterization of japonica rice lines carrying the brown planthopper-resistance genes *Bph12* and *Bph6*. Theor Appl Genet.

[CR36] Ren J, Gao F, Wu X, Lu X, Zeng L, Lv J, Su X, Luo H, Ren G (2016). *Bph32*, a novel gene encoding an unknown SCR domain-containing protein, confers resistance against the brown planthopper in rice. Sci Reports.

[CR37] Sogawa K, Liu GJ, Shen JH (2003). A review on the hyper-susceptibility of Chinese hybrid rice to insect pests. Chin J Rice Sci.

[CR38] Srinivasan TS, Almazan MLP, Bernal CC (2015). Current utility of the *BPH25* and *BPH26* genes and possibilities for further resistance against plant- and leafhoppers from the donor cultivar ADR52. Appl Entomol Zool.

[CR39] Su CC, Zhai HQ, Wang CM, Sun LH, Wan JM (2006). SSR mapping of brown planthopper resistance gene *Bph9* in Kaharamana, an indica rice (Oryza Sativa L.). Acta Genet Sin.

[CR40] Tamura Y, Hattori M, Yoshioka H, Yoshioka M, Takahashi A, Wu JZ, Sentoku N, Yasui H (2014). Map-based cloning and characterization of a brown planthopper resistance gene BPH26 from Oryza Sativa L. Ssp. Indica cultivar ADR52. Sci Reports.

[CR41] Tanaka K, Endo S, Kazano H (2000). Toxicity of insecticides to predators of rice planthoppers: spiders, the mirid bug and the dryinid wasp. Appl Entomol Zool.

[CR42] Wan BL, Zha ZP, Li JB, Xia MY, Du XS, Lin YJ, Yin DS (2014). Development of elite rice restorer lines in the genetic background of R022 possessing tolerance to brown planthopper, stem borer, leaf folder and herbicide through marker-assisted breeding. Euphytica.

[CR43] Wang HB, Ye ST, Mou TM (2016). Molecular breeding of Rice restorer lines and hybrids for Brown Planthopper (BPH) resistance using the *Bph14* and *Bph15* genes. Rice.

[CR44] Wang Y, Cao LM, Zhang YX, Cao CX, Liu F, Huang FK, Qiu YF, Li RB, Lou XJ (2015). Map-based cloning and characterization of *BPH29*, a B3 domain-containing recessive gene conferring brown planthopper resistance in rice. J Exp Bot.

[CR45] Watanabe T, Kitagawa H (2000). Photosynthesis and translocation of assimilates in rice plants following phloem feeding by the planthopper *Nilaparvata lugens* (Homoptera: Delphacidae). J Econ Entomol.

[CR46] Xia M, Wan NB, Li XY, Zha Z, Du X, Qi H (2010). Breeding and application of new quality medimun indica hybrid rice (in Chinese with English abstract). Hybrid Rice.

[CR47] Yang HY, Ren X, Weng QM, Zhu LL, He GC (2002). Molecular mapping and genetic analysis of a rice brown planthopper (*Nilaparvata lugens* Stål) resistance gene. Hereditas.

[CR48] Yeo US, Shon JK (2001). Linkage analysis between some agronomic traits and resistance gene to brown planthopper in rice. Korean J Plant Breed.

[CR49] Yu H, Xie W, Li J, Zhou F, Zhang Q (2014). A whole-genome SNP array (RICE6K) for genomic breeding in RICE. Plant Biotechnol J.

[CR50] Zhao Y, Huang J, Wang Z et al (2016) Allelic diversity in an NLR gene *BPH9* enables rice to combat planthopper variation. Proc Natl Acad Sci U S A 113:12850-12855 10.1073/pnas.1614862113 PMC5111712 27791169

[CR51] Zhou L, Chen ZJ, Lang XY, Du B, Liu K, Yang GC, Hu G, Li SH, He GC, You AQ (2013). Development and validation of a PCR-based functional marker system for the brown planthopper resistance gene Bph14 in rice. Breeding Sci.

[CR52] Zhu R, Huang W, Hu J, Liu W, Zhu Y (2013). Breeding of new sterile line Luohong 4A of Honglian type hybrid rice (in Chinese with English abstract). J Wuhan Univ (Nat Sci Ed).

[CR53] Zhu R, Huang W, Hu J, Liu W, Zhu Y (2013). Breeding and utilization of hybrid rice Liangyou234 and NMS line Bph68S resistance to brown planthopper (in Chinese with English abstract). J Wuhan Univ (Nat Sci Ed).

