# Supplementary figures and images for: Marker assisted pyramiding of Bph6 and Bph9 into elite restorer line 93–11 and development of functional marker for Bph9
Source: Rice (N Y). 2017 Dec 28;10:51. doi: 10.1186/s12284-017-0194-x (PMC5745207; doi:10.1186/s12284-017-0194-x)

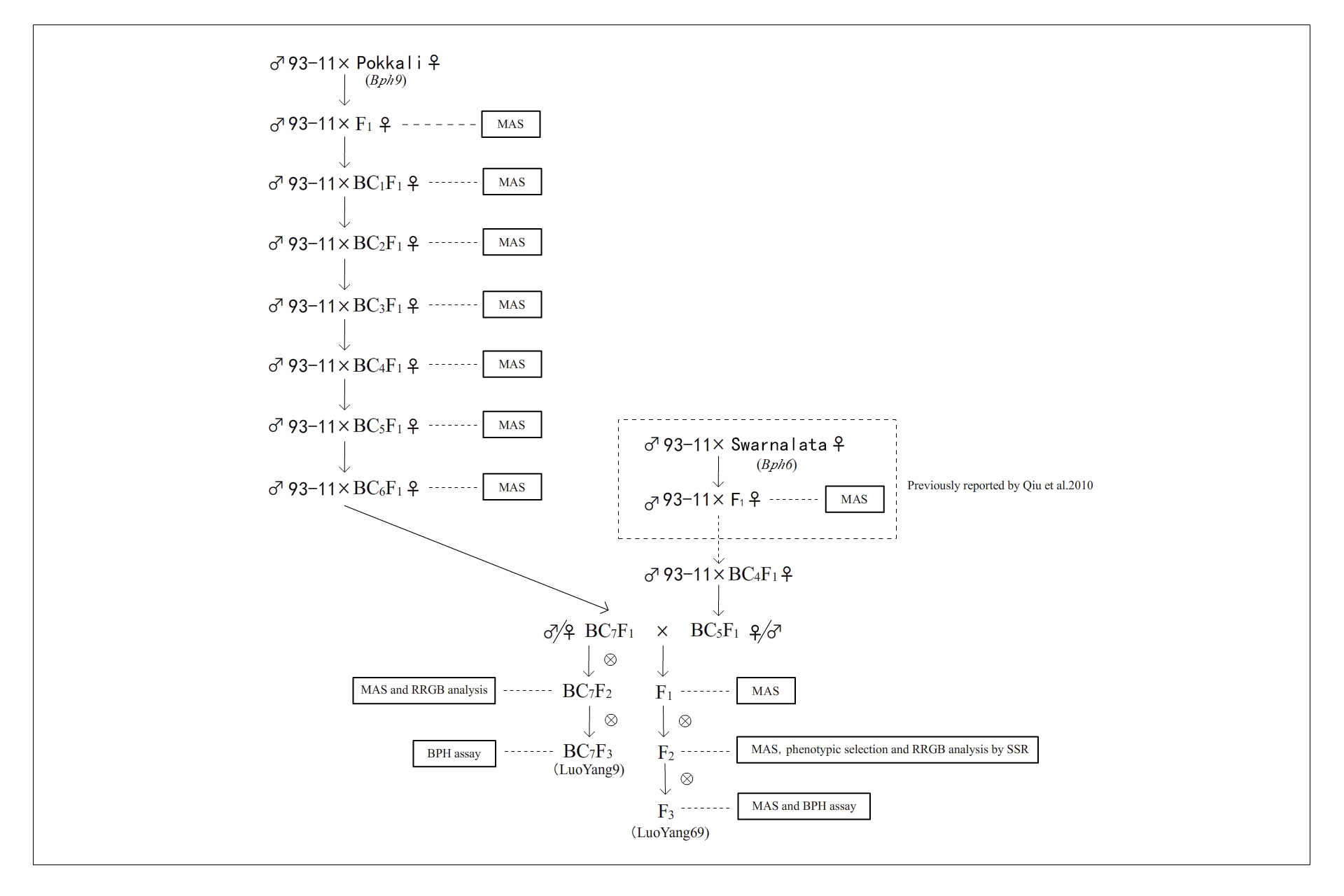

Supplement: Supplementary file 1 — Strategy used to pyramid Bph6 and Bph9 in 93–11 genetic background. Pokkali and Swarnalata are the resistance donors of Bph9 and Bph6, respectively. The resulting Bph6 and Bph9 pyramided line in 93–11 genetic background is designated as LuoYang69. (TIFF 330 kb) [file 12284_2017_194_MOESM1_ESM.tif]

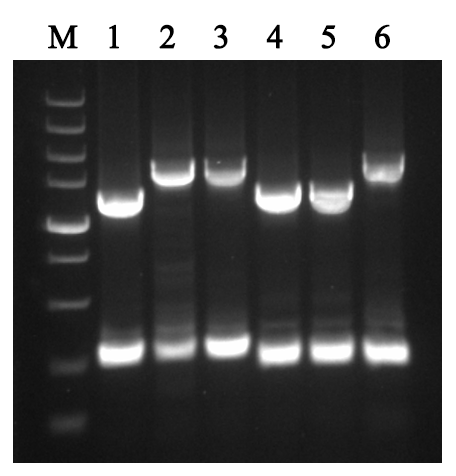

Supplement: Supplementary file 2 — PCR analysis of LuoYang69 with molecular markers InD2 (for Bph9) and H (for Bph6). M: DL5000; 1 to 6: 93–11, Swarnalata, NIL-Bph6, Pokkali, NIL-Bph9, LuoYang69. (TIFF 2603 kb) [file 12284_2017_194_MOESM2_ESM.tif]
